# Effects of pay for performance on risk incidence of infection and of revision after total knee arthroplasty in type 2 diabetic patients: A nationwide matched cohort study

**DOI:** 10.1371/journal.pone.0206797

**Published:** 2018-11-02

**Authors:** Yi-Shiun Tsai, Pei-Tseng Kung, Ming-Chou Ku, Yeuh-Hsin Wang, Wen-Chen Tsai

**Affiliations:** 1 Department of Orthopedics, Feng Yuan Hospital, Ministry of Health and Welfare, Taichung, Taiwan, ROC; 2 Department of Health Services Administration, China Medical University, Taichung, Taiwan, ROC; 3 Department of Healthcare Administration, Asia University, Taichung, Taiwan, ROC; 4 Department of Medical Research, China Medical University Hospital, China Medical University, Taichung, Taiwan, R.O.C; 5 Department of Orthopedics, Chang Bing Show Chwan Memorial Hospital, Changhua, Taiwan, ROC; University of Michigan, UNITED STATES

## Abstract

As the world’s population ages, the number of people receiving total knee arthroplasty (TKA) has been on the rise. Although patients with diabetes mellitus are known to face greater risks of TKA postoperative infection and revision TKA owing to diabetic complications, studies on whether such patients’ participation in pay for performance (P4P) programs influences the incidence rates of TKA postoperative infection or revision TKA are still lacking. This study examined the 2002–2012 data of Taiwan’s National Health Insurance Research Database to conduct a retrospective cohort analysis of diabetic patients over 50 years old who have received TKA. To reduce any selection bias between patients joining and not joining the P4P program, propensity score matching was applied. The Cox proportional hazards model was used to examine the influence of the P4P program on TKA postoperative infection and revision TKA, and the results indicate that joining P4P lowered the risk of postoperative infection (HR = 0.91, 95% CI: 0.77–1.08), however, which was not statistically significant, and significantly lowered the risk of revision TKA (HR = 0.53, 95% CI: 0.39–0.72). Being younger and male, having multiple comorbid conditions or greater diabetic severity, receiving care at regional or public hospitals, and not having a diagnosis of degenerative or rheumatoid arthritis were identified as factors for higher risk of TKA postoperative infection for patients with diabetes. As for the risk of revision TKA, postoperative infection and being younger were identified as factors for a significantly higher risk (*p* < 0.05).

## Introduction

Total knee arthroplasty (TKA) is often performed in the late stage of knee arthritis [1]. As the world’s population ages, the number of people receiving this surgical treatment has been on the rise. By 2010, more than 600,000 people underwent TKA in the United States, and the number is rising every year. Medicare has predicted that from 2007 to 2035, the number of TKA cases will see a 673% increase, reaching 3.48 million cases [2]. In Taiwan, the frequency of TKA was 28.5 cases per 100,000 people in 1998 and 56.8 cases per 100,000 people in 2009, which is a 99.1% increase; at this rate, the number is expected to see a 508.2% increase by 2030 [3].

With the increase in TKA cases, cases of postoperative infection and revision TKA (i.e., the replacement of the previously implanted artificial knee joint) have also become increasingly common [4, 5]. Although TKA operations are generally successful, inevitably some patients require revisions owing to complications such as postoperative infection. In most cases, patients undergoing revision TKA experience increased pain, and lowered prosthetic functionality and service life than primary TKA [6]. Moreover, for cases of prosthetic infection, the site must usually be cleaned numerous times before revision TKA, which is not only detrimental to patients’ quality of life but also increases their medical expenses [7].

Diabetes mellitus has been found to significantly enhance the risk of severe osteoarthritis, thus conferring a higher probability of requiring TKA [8, 9]. Furthermore, because diabetes weakens the immune system, patients with diabetes also face a higher postoperative infection rate after TKA, which must be countered by preoperative well-managed blood glucose control [10].

The concept of “pay for performance,” or P4P, has been widely applied around the world, such as in the United Kingdom and in Australia [11–13]. Essentially, it converts the role of health insurance from cost-based purchasing to quality-based purchasing, and by connecting financial incentives with medical quality, medical institutions and physicians are encouraged to provide high quality, low cost, and comprehensive medical services. In Taiwan, the National Health Insurance Administration initiated a P4P program for diabetes in November 2001. By 2009, 27.56% of Taiwanese patients with diabetes participated in the Diabetes P4P programs [14]. Under this program, multifunctional healthcare teams were formed by professional medical personnel including physicians, nurses, nutritionists, medical laboratory technologists, and health education specialists to periodically provide extensive, continuous, and patient-centered medical services that cover the diagnosis, examination, health education, and tracking of patients with diabetes. The performance of such teams is gauged by four major indicators, namely the completeness of patients’ follow-up records, percentage of patients with glycated hemoglobin <7%, percentage of patients with glycated hemoglobin >9.5%, and percentage of patients with low-density lipoprotein >130 mg/L. Financial rewards are provided to the medical personnel as incentives to provide comprehensive and continuous care for the patients and improve the effectiveness of their treatment [15].

P4P programs in general have been found to improve diabetic outcome measures, but not all P4P programs are equal; some are more effective than others[16–18]. For the patients, the P4P program enables them to receive comprehensive and continuous medical care from multifunctional healthcare teams, which raises the rate of patients seeking continual medical attention and reduces the risks of amputation and mortality [19, 20] Numerous studies have also confirmed that joining the P4P program improves the patients’ blood glucose and glycated hemoglobin [21–23].

Despite the abundance of studies discussing the influence of diabetes on TKA postoperative infection and revision, no study has examined the influence after the intervention of the P4P program. Therefore, the present study examined the influence of joining the P4P program, in an effort to provide a reference for medical policies on diabetes and to reduce instances of TKA postoperative infection and revision through patients’ effective management of their diabetes conditions.

## Material and methods

### Study subjects

**Fig 1:**
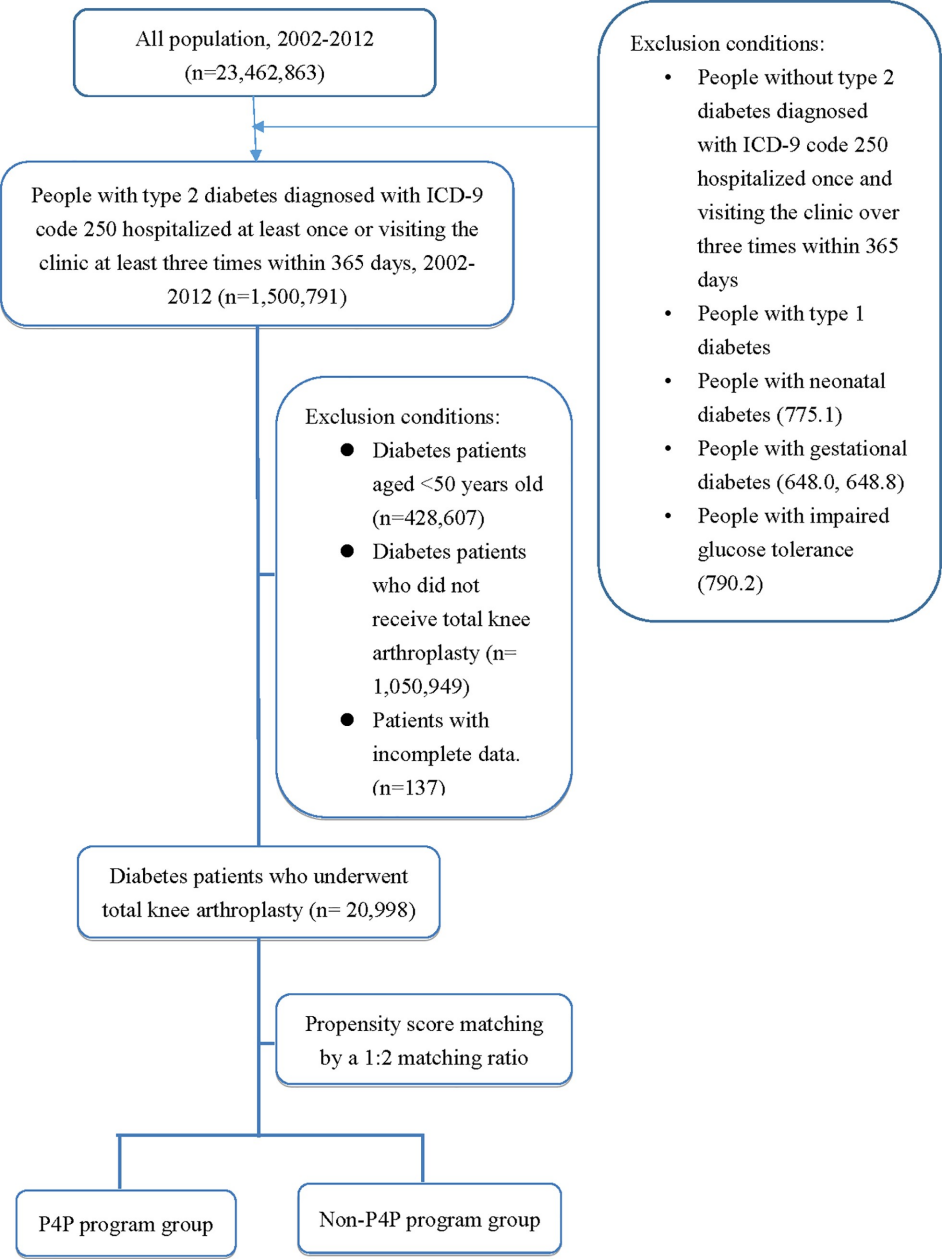
Flow chart of participant collection. The diabetes patients, having undergone TKA, were divided into the P4P group and non-P4P group by propensity score matching.

The study sample was drawn from the 2002–2013 data in the National Health Insurance (NHI) Research Database. The inclusion criteria for patients were a diagnosis of diabetes, having undergone TKA, and being more than 50 years of age. “Having diabetes” was defined as having been hospitalized at least once or visiting the clinic at least three times within 365 days because of type 2 diabetes (ICD-9-CM 250.XX or A-code A181) as the primary or secondary diagnosis [24]. Also, the reception of TKA was defined as being hospitalized for the surgical procedure called “total knee arthroplasty” (TKR order codes 64164B, 97805K, 97806A, or 97807B). Because this study focused on type 2 diabetes, patients with type 1 diabetes, neonatal diabetes (775.1), gestational diabetes (648.0, 648.8), and impaired glucose tolerance (790.2) were excluded. The patients were further divided into two groups depending on whether they had joined the P4P program. The flow chart of study participant collection was shown as Fig 1.

### Data sources

This is a retrospective cohort study using secondary data taken from the NHI database established by the National Health Research Institute. The NHI is compulsory health insurance for Taiwan residents; it was launched in 1995 and 99.85% of the population was enrolled as of 2012 [21]. Specifically, this study extracted its data from the 2001–2013 diabetes entries in the NHI database, which, beside patient demographics, contained information such as participation in the P4P program and receipt of TKA.

### Descriptions of variables

In this study, the independent variable was whether a patient had joined the P4P program. This was determined by whether the code “E4” was marked in their medical records. The dependent variables were TKA postoperative infection and revision TKA. Patients whose medical records included the following ICD-9-CM codes within 1 year of receiving TKA were defined as having TKA postoperative infection: 711.00 or 711.06 (pyogenic arthritis); 998.3 or 998.5 (other complications from surgical procedures); 730.25, 730.26, or 730.28 (osteomyelitis); or 996.60, 996.66, or 996.67 (infection and inflammatory reaction due to internal prosthetic device implant and graft) [25]. Revision TKA was defined as undergoing the removal and partial/complete replacement of the prosthetic device (TKR order code: 64202B) [26, 27].

The control variables included patient demographic characteristics, economic factors, environmental factors, health status, characteristics of their primary medical institution, the service volume of each patient’s primary surgeon, and their primary diagnosis for TKA.

The demographic characteristics included gender and age, and economic status was measured by monthly income. Environmental status concerns the degree of urbanization of the patient’s area of residence, which is divided into seven levels (1 being the most urbanized and 7 the least) [28]. The patient’s health status was based on the severity of comorbid conditions and complications. The severity of comorbid conditions was measured by Deyo’s version of the Charlson Comorbidity Index (CCI), which divides comorbid conditions into 17 types [29]. After converting the ICD-9-CM codes of the patient’s primary and secondary diagnosis into weighed numerical points, Deyo’s CCI score could be obtained from their sum [29], with a higher CCI score indicating greater severity of the comorbid condition. The severity of complications was measured by the Diabetes Complications Severity Index (DCSI), which was the sum of the patient’s primary and secondary diagnostic codes (ICD-9-CM) graded by the presence and severity of seven diabetic complications (cardiovascular disease, nephropathy, retinopathy, peripheral vascular disease, stroke, neuropathy, and endocrinopathy), as proposed by Young et al., with the highest score being 13 points [30]. A higher DCSI score indicated a greater severity of diabetes. Both the CCI and DCSI scores were calculated based on the individual patients’ medical records for 1 year before they met the criteria for inclusion in this study.

The primary medical institution here refers to the medical institution where the patient received TKA. Such institutions were divided by status into medical centers, regional hospitals, and district hospitals, and by ownership into public hospitals and private hospitals [31]. The medical institutions were also categorized by service volume, which was divided into low (<25%), moderate (25–75%), and high (>75%) levels using the quartile method. Likewise, the service volume of the patient’s primary surgeon, which refers to how many times that particular surgeon performed TKA in the year the patient received the surgery, was divided into low (<25%), moderate (25–75%), and high (>75%) levels using the quartile method. The primary diagnosis refers to the diagnosis that caused the patient to be admitted to a medical institution for TKA; this category included degenerative arthritis (ICD-9-CM code: 715), rheumatoid arthritis (ICD-9-CM code: 714), and other diagnostic results.

### Statistical analysis

First, descriptive statistics were applied to determine the distribution of patients and the various variables, followed by the chi-square test to determine whether joining the P4P program made a difference between the patients in terms of said variables. To reduce any selection bias between patients regarding joining and not joining the program, propensity score matching was applied [22, 23]. Specifically, logistic regression analysis to estimate the probability (i.e., the propensity score, 0–1), according to which the patients were divided into the “P4P program group” and the “non-P4P program group” by a 1:2 matching ratio.

The Cox proportional hazards model was then employed to explore the influence of joining the P4P program, as well as other related factors, on TKA postoperative infection and revision TKA. The results were presented as hazard ratios (HRs) and 95% confidence intervals (CIs). In addition, the Kaplan–Meier estimator was used to predict the survival duration of the prosthetic device, and the log-rank test was used to determine if joining the P4P program was correlated with a statistically significant risk of revision TKA for patients.

This study used SAS version 9.4 (SAS Institute Inc., Cary, NC, USA) for the data processing and analysis, and all of the tests were two-tailed. Statistical significance was defined as *p* < 0.05. The study has been approved by the Institutional Review Board of author’s organization (IRB No.: CMUH 103-REC3-109).

## Results

A total of 20,988 patients who met the inclusion criteria constituted the population of this study. Statistically significant differences in demographics and economic, environmental, and health conditions were observed between P4P and non-P4P patients (*p* < 0.05). In studies concerning the influence of the P4P program on patients with TKA postoperative infection, postoperative infection was defined as infection occurring within one year of TKA; hence, patients’ postoperative statuses were followed-up for one year. Accordingly, a total of 4,337 P4P patients were found in the present study. Applying propensity score matching to the patients at a 1:2 ratio generated 8,562 who did not join the P4P program, and 4,281 patients who did join, totaling 12,843 patients. After the matching, however, whether patients had joined the P4P program or not was not associated with any significant differences (*p* > 0.05) in demographics, economic, environmental, or health conditions (Table 1).

**Table 1:** Comparisons of study subjects in TKA postoperative infections study after propensity score matching for P4P participating status.

Variable	Before matching							After matching (ratio = 2:1)						
	Total		Non-P4P		P4P		*p*	Total		Non-P4P		P4P		*p*
	Cases	%	Cases	%	Cases	%		Cases	%	Cases	%	Cases	%	
Total	20988	100	16651	79.34	4337	20.66		12843	100	8562	66.67	4281	33.33	
Gender							0.750							0.150
Female	15749	75.04	12486	79.28	3263	20.72		9757	75.97	6538	67.01	3219	32.99	
Male	5239	24.96	4165	79.5	1074	20.5		3086	24.03	2024	65.59	1062	34.41	
Age (years)							<0.001							0.996
50–54	1498	7.14	1067	71.23	431	28.77		1197	9.32	795	66.42	402	33.58	
55–64	7233	34.46	5447	75.31	1786	24.69		5277	41.09	3518	66.67	1759	33.33	
65–74	9412	44.84	7633	81.1	1779	18.9		5351	41.66	3572	66.75	1779	33.25	
≥75	2845	13.56	2504	88.01	341	11.99		1018	7.93	677	66.5	341	33.5	
Monthly income (NT$)							0.038							0.392
Low-income households	129	0.61	105	81.4	24	18.6		52	0.4	28	53.85	24	46.15	
≤17280	804	3.83	625	77.74	179	22.26		500	3.89	326	65.2	174	34.8	
17281–22080	15456	73.64	12267	79.37	3189	20.63		9525	74.16	6373	66.91	3152	33.09	
22081–28800	1652	7.87	1311	79.36	341	20.64		1049	8.17	710	67.68	339	32.32	
28801–36300	928	4.42	703	75.75	225	24.25		612	4.77	397	64.87	215	35.13	
36301–45800	995	4.74	810	81.41	185	18.59		533	4.15	350	65.67	183	34.33	
≥45801	1024	4.88	830	81.05	194	18.95		572	4.45	378	66.08	194	33.92	
Degree of urbanization in area of residence														
							<0.001							1
Level 1	4551	21.68	3672	80.69	879	19.31		2628	20.46	1749	66.55	879	33.45	
Level 2	5388	25.67	4253	78.93	1135	21.07		3373	26.26	2260	67	1113	33	
Level 3	3281	15.63	2636	80.34	645	19.66		1901	14.8	1266	66.6	635	33.4	
Level 4	3908	18.62	3035	77.66	873	22.34		2577	20.07	1714	66.51	863	33.49	
Level 5	912	4.35	753	82.57	159	17.43		477	3.71	318	66.67	159	33.33	
Level 6	1755	8.36	1355	77.21	400	22.79		1149	8.95	763	66.41	386	33.59	
Level 7	1193	5.68	947	79.38	246	20.62		738	5.75	492	66.67	246	33.33	
CCI score							0.011							0.370
0	7121	33.93	5592	78.53	1529	21.47		4391	34.19	2910	66.27	1481	33.73	
1	10923	52.04	8669	79.36	2254	20.64		6843	53.28	4597	67.18	2246	32.82	
≥2	2944	14.03	2390	81.18	554	18.82		1609	12.53	1055	65.57	554	34.43	
DCSI score							<0.001							0.992
0	13669	65.13	11112	81.29	2557	18.71		7655	59.6	5100	66.62	2555	33.38	
1	3826	18.23	2793	73.00	1033	27.00		2956	23.02	1973	66.75	983	33.25	
≥2	3493	16.64	2746	78.61	747	21.39		2232	17.38	1489	66.71	743	33.29	

Note: This table shows the chi-square test results

**Fig 2:**
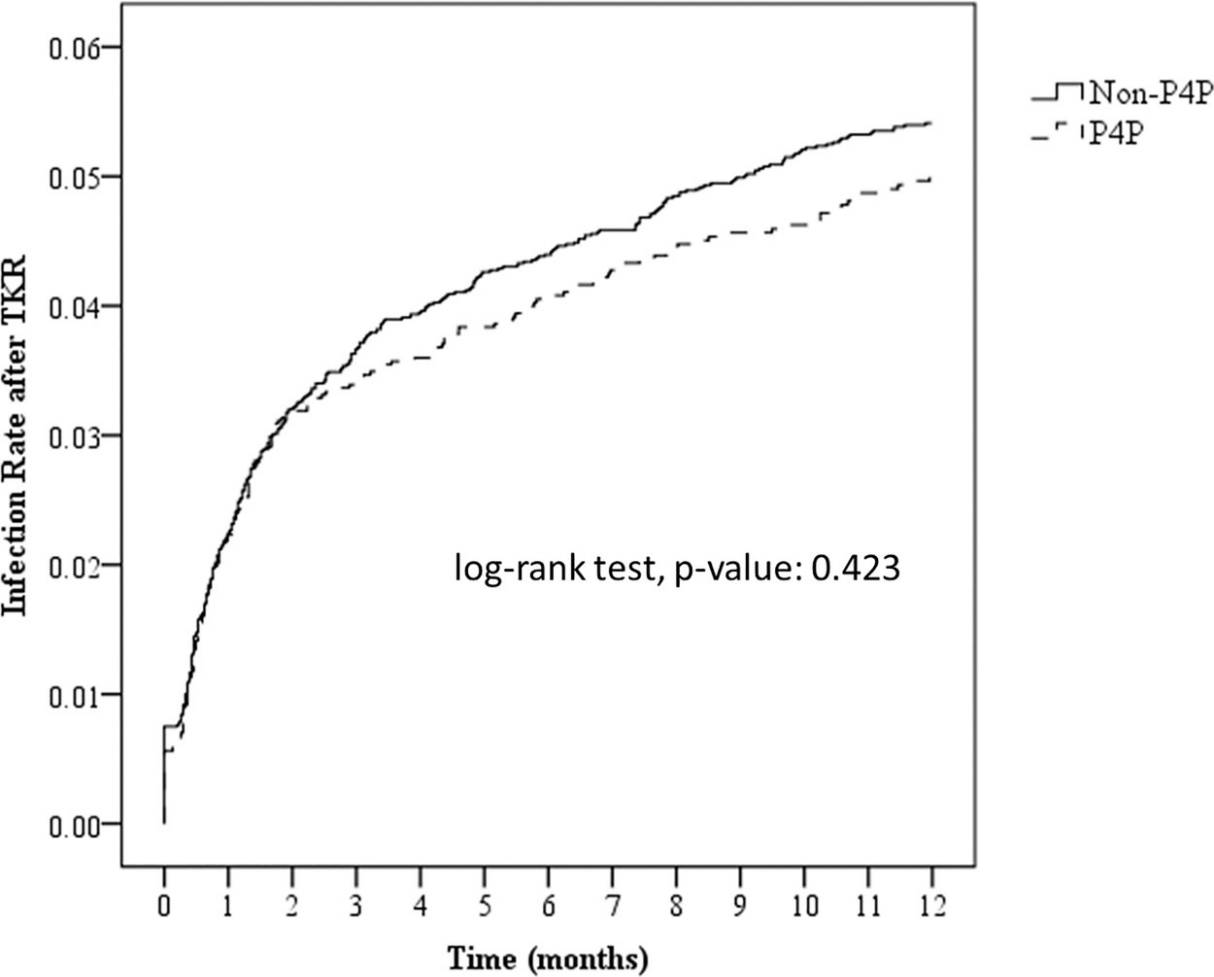
Cumulative risk curves of TKA postoperative infection for P4P and non-P4P patients. Joining the P4P program is associated with a tendency of lowered risk, but this result is not statistically significant (*p* = 0.423).

According to Table 2, TKA postoperative infection occurred in 4.92% of the patients overall, 5.05% of non-P4P patients, and 4.67% of P4P patients. This suggests that the occurrence was noticeably less frequent among P4P patients. Moreover, the cumulative risk curves in Fig 2 indicate that non-P4P patients faced a higher cumulative risk of postoperative infection. However, the association between participation in the P4P program and TKA postoperative infection did not attain statistical significance in the bivariate analysis (*p* = 0.423). Other variables did attain statistical significance with TKA postoperative infection (*p* < 0.05): gender, age, monthly income, degree of urbanization in area of residence, severity of comorbid conditions (CCI), severity of diabetic complications (DCSI), the service volume of the surgeon, the status of the primary medical institution, the ownership of the primary medical institution, and the primary diagnosis for TKA.

**Table 2:** Factors associated with TKA postoperative infections in patients with diabetes.

Variable	Total		Not infected		Infected		*p*^a^	Adjusted			
	Cases	%	Cases	%	Cases	%		HR	95%CI	*p*^b^
Total	12843	100	12211	95.08	632	4.92					
Joined P4P program							0.423				
No (Ref.)	8562	66.67	8130	94.95	432	5.05					
Yes	4281	33.33	4081	95.33	200	4.67		0.91	0.77	1.08	0.277
Gender							<0.001				
Female (Ref.)	9757	75.97	9338	95.71	419	4.29					
Male	3086	24.03	2873	93.1	213	6.9		1.51	1.27	1.78	< .0001
Age (years)							0.016				
50–54 (Ref.)	1197	9.32	1116	93.23	81	6.77					
55–64	5277	41.09	5030	95.32	247	4.68		0.73	0.56	0.94	0.013
65–74	5351	41.66	5098	95.27	253	4.73		0.7	0.54	0.9	0.006
≥75	1018	7.93	967	94.99	51	5.01		0.65	0.46	0.94	0.020
Monthly income (NT$)							0.001				
Low-income households (Ref.)	52	0.4	43	82.69	9	17.31					
≤17280	500	3.89	481	96.2	19	3.8		0.37	0.16	0.83	0.016
17281–22080	9525	74.16	9039	94.9	486	5.1		0.43	0.22	0.85	0.014
22081–28800	1049	8.17	1004	95.71	45	4.29		0.4	0.19	0.83	0.015
28801–36300	612	4.77	585	95.59	27	4.41		0.42	0.19	0.9	0.026
36301–45800	533	4.15	510	95.68	23	4.32		0.43	0.19	0.93	0.033
≥45801	572	4.45	549	95.98	23	4.02		0.4	0.18	0.89	0.024
Degree of urbanization in area of residence							0.007				
Level 1 (Ref.)	2628	20.46	2528	96.19	100	3.81					
Level 2	3373	26.26	3215	95.32	158	4.68		1.12	0.87	1.45	0.371
Level 3	1901	14.8	1798	94.58	103	5.42		1.35	1.02	1.78	0.036
Level 4	2577	20.07	2452	95.15	125	4.85		1.16	0.88	1.52	0.301
Level 5	477	3.71	443	92.87	34	7.13		1.75	1.17	2.61	0.006
Level 6	1149	8.95	1083	94.26	66	5.74		1.31	0.95	1.82	0.100
Level 7	738	5.75	692	93.77	46	6.23		1.42	0.99	2.04	0.056
CCI score							<0.001				
0 (Ref.)	4391	34.19	4231	96.36	160	3.64					
1	6843	53.28	6476	94.64	367	5.36		1.34	1.11	1.62	0.002
≥2	1609	12.53	1504	93.47	105	6.53		1.44	1.11	1.87	0.006
DCSI score							<0.001				
0 (Ref.)	7655	59.6	7315	95.56	340	4.44					
1	2956	23.02	2817	95.3	139	4.7		1.07	0.88	1.31	0.494
≥2	2232	17.38	2079	93.15	153	6.85		1.34	1.09	1.64	0.005
Service volume (surgeon)							<0.001				
Low (Ref.)	770	6	713	92.6	57	7.4					
Moderate	3436	26.75	3235	94.15	201	5.85		0.8	0.59	1.09	0.163
High	8637	67.25	8263	95.67	374	4.33		0.63	0.46	0.86	0.004
Service volume (medical institution)							0.094				
Low (Ref.)	313	2.44	292	93.29	21	6.71					
Moderate	3005	23.4	2841	94.54	164	5.46		0.92	0.57	1.5	0.737
High	9525	74.16	9078	95.31	447	4.69		1.11	0.68	1.81	0.691
Status of medical institution							<0.001				
Medical center (Ref.)	4431	34.5	4276	96.5	155	3.5					
Regional hospital	4795	37.34	4492	93.68	303	6.32		1.9	1.54	2.34	< .0001
District hospital	3617	28.16	3443	95.19	174	4.81		1.52	1.2	1.92	0.001
Ownership of medical institution							<0.001				
Public (Ref.)	4368	34.01	4113	94.16	255	5.84					
Private	8475	65.99	8098	95.55	377	4.45		0.67	0.57	0.78	< .0001
Primary diagnosis						<0.001				
Degenerative arthritis (Ref.)	12506	97.38	11916	95.28	590	4.72					
Rheumatoid arthritis	87	0.68	82	94.25	5	5.75		1.08	0.45	2.63	0.860
Others	250	1.95	213	85.2	37	14.8		2.86	2.04	4.01	< .0001
Year							0.223				
2002 (Ref.)	1792	13.95	1701	94.92	91	5.08					
2003	1674	13.03	1592	95.1	82	4.90		0.98	0.73	1.33	0.915
2004	1674	13.03	1591	95.04	83	4.96		0.99	0.73	1.33	0.940
2005	1543	12.01	1464	94.88	79	5.12		1.04	0.77	1.41	0.780
2006	1328	10.34	1253	94.35	75	5.65		1.13	0.83	1.54	0.436
2007	1222	9.51	1158	94.76	64	5.24		1.05	0.76	1.45	0.770
2008	1126	8.77	1082	96.09	44	3.91		0.80	0.56	1.15	0.233
2009	956	7.44	912	95.4	44	4.60		0.97	0.68	1.4	0.873
2010	723	5.63	701	96.96	22	3.04		0.62	0.39	0.99	0.046
2011	543	4.23	511	94.11	32	5.89		1.19	0.79	1.78	0.406
2012	256	1.99	240	93.75	16	6.25		1.39	0.81	2.37	0.231

Note: *p* ^a^ represents the log-rank test results

*p*^b^ represents the Cox proportional hazard model results

Table 2 shows the results of using the Cox proportional hazards model to examine the risk factors related to TKA postoperative infection for patients with diabetes. When all of the other variables were controlled, the risk faced by P4P patients was found to be 0.91 times that of non-P4P patients (HR = 0.91, 95% CI: 0.77–1.08). This, however, was not statistically significant. The risk facing male patients was 1.51 times higher than that facing female patients (95% CI: 1.27–1.78). With the age group of 50–54 years set as the reference group, the results indicate that the risk dropped for every 10 years of increased age; for patients of ages ≥75 years, the risk was only 0.65 times that of the reference group (95% CI: 0.46–0.94).

Regarding the severity of comorbid conditions and diabetic complications, a higher CCI score signified a greater risk of TKA postoperative infection. Therefore, for CCI ≥2, the risk was 1.44 times higher than for CCI = 0 (HR = 1.44, 95% CI: 1.11–1.87). Likewise, for DCSI ≥2, the risk was 1.34 times higher than for DCSI = 0 (95% CI: 1.09–1.64). Moreover, the risk was higher in regional hospitals than in medical centers (HR = 1.90, 95% CI: 1.54–2.34), and lower in private hospitals than in public hospitals (HR = 0.67, 95% CI: 0.57–0.78). Concerning primary diagnosis, the risk was higher for patients undergoing TKA for conditions other than degenerative or rheumatoid arthritis (HR = 2.86, 95% CI: 2.04–4.01).

For revision TKA, because the duration from the start to the conclusion of observation or until revision TKA was performed could be as long as 11 years, 5,506 people were noted to have joined the P4P program during that time. By applying propensity score matching on these cases at a 1:2 ratio based on whether they joined the P4P program, 15,402 TKA patients were identified, of whom 10,268 did not join the P4P program and 5,134 did (Table 3). Before matching, the two groups of patients exhibited significant differences (*p* < 0.05) in age as well as economic, environmental, and health conditions, but after matching, no significant differences (*p* > 0.05) were found (Table 3).

**Table 3:** Comparisons of study subjects in revision TKA study after propensity score matching for P4P participating status.

Variable	Before matching							After matching (ratio = 2:1)						
	Total		Non-P4P		P4P		*p*	Total		Non-P4P		P4P		*p*
	Cases	%	Cases	%	Cases	%		Cases	%	Cases	%	Cases	%	
Total	20988	100	15482	73.77	5506	26.23		15402	100	10268	66.67	5134	33.33	
Gender							0.278							0.695
Female	15749	75.04	11587	73.57	4162	26.43		11665	75.74	7787	66.76	3878	33.24	
Male	5239	24.96	3895	74.35	1344	25.65		3737	24.26	2481	66.39	1256	33.61	
Age (years)							< .0001							0.978
50–54	1498	7.14	978	65.29	520	34.71		1195	7.76	792	66.28	403	33.72	
55–64	7233	34.46	5029	69.53	2204	30.47		5970	38.76	3991	66.85	1979	33.15	
65–74	9412	44.84	7082	75.24	2330	24.76		6887	44.71	4587	66.60	2300	33.40	
≥75	2845	13.56	2393	84.11	452	15.89		1350	8.77	898	66.52	452	33.48	
Monthly income (NT$)							0.033							0.584
Low-income households	129	0.61	97	75.19	32	24.81		64	0.42	35	54.69	29	45.31	
≤17280	804	3.83	581	72.26	223	27.74		613	3.98	409	66.72	204	33.28	
17281–22080	15456	73.64	11377	73.61	4079	26.39		11486	74.57	7676	66.83	3810	33.17	
22081–28800	1652	7.87	1220	73.85	432	26.15		1206	7.83	803	66.58	403	33.42	
28801–36300	928	4.42	659	71.01	269	28.99		688	4.47	456	66.28	232	33.72	
36301–45800	995	4.74	762	76.58	233	23.42		653	4.24	428	65.54	225	34.46	
≥45801	1024	4.88	786	76.76	238	23.24		692	4.49	461	66.62	231	33.38	
Degree of urbanization in area of residence							< .0001							0.987
Level 1	4551	21.68	3462	76.07	1089	23.93		3162	20.53	2103	66.51	1059	33.49	
Level 2	5388	25.67	3985	73.96	1403	26.04		3974	25.8	2661	66.96	1313	33.04	
Level 3	3281	15.63	2464	75.10	817	24.90		2265	14.71	1520	67.11	745	32.89	
Level 4	3908	18.62	2774	70.98	1134	29.02		3099	20.12	2053	66.25	1046	33.75	
Level 5	912	4.35	701	76.86	211	23.14		619	4.02	414	66.88	205	33.12	
Level 6	1755	8.36	1251	71.28	504	28.72		1281	8.32	857	66.90	424	33.10	
Level 7	1193	5.68	845	70.83	348	29.17		1002	6.51	660	65.87	342	34.13	
CCI score							< .0001							0.380
0	7121	33.93	5170	72.60	1951	27.40		5293	34.37	3539	66.86	1754	33.14	
1	10923	52.04	8041	73.62	2882	26.38		8227	53.42	5501	66.87	2726	33.13	
≥2	2944	14.03	2271	77.14	673	22.86		1882	12.22	1228	65.25	654	34.75	
DCSI score							< .0001							0.839
0	13669	65.13	10313	75.45	3356	24.55		9990	64.86	6675	66.82	3315	33.18	
1	3826	18.23	2581	67.46	1245	32.54		2763	17.94	1830	66.23	933	33.77	
≥2	3493	16.64	2588	74.09	905	25.91		2649	17.2	1763	66.55	886	33.45	

Note: This table presents chi-square test results

**Fig 3:**
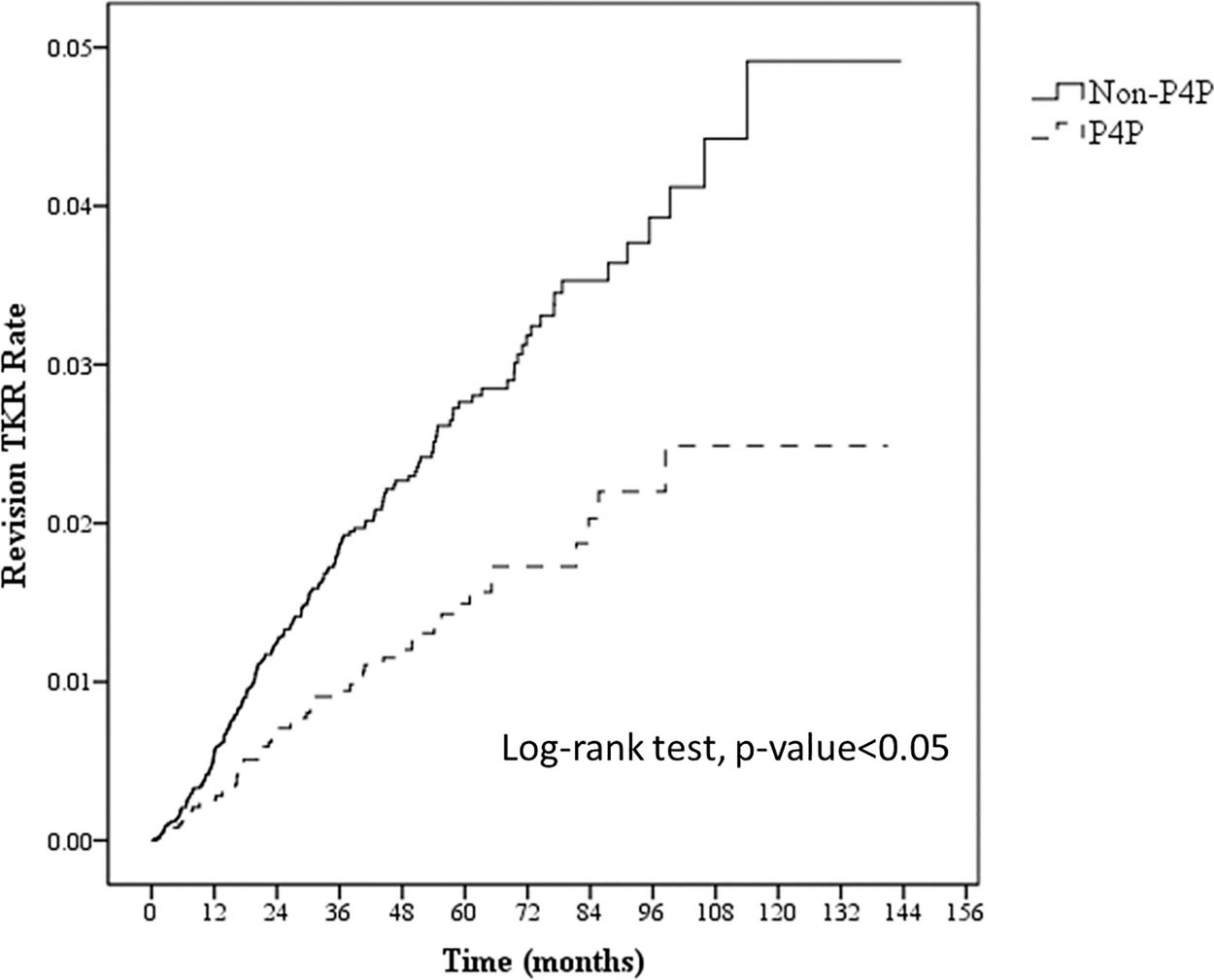
Cumulative risk curves of revision TKA for P4P and non-P4P patients. Joining the P4P program is associated with a tendency of significantly lowered risk (log-rank test; *p* < 0.05).

The annual incidence rate of revision TKA among patients with diabetes averaged 4.54 incidents per thousand people overall. The rate was 5.40 among non-P4P patients, which was significantly greater than the 2.92 for the P4P patients (Table 4). Furthermore, the curves for cumulative risk in Fig 3 indicate that non-P4P patients faced a greater cumulative risk of revision TKA than the P4P patients.

**Table 4:** Factors associated with revision TKA in patients with diabetes.

Variable	Total	No revision TKA	Revision TKA	Per thousand per year	Adjusted †			
					HR	95%CI		*p*
Total	15402	15162	240	4.54				
Joined P4P program								
No	10268	10082	186	5.40	1			
Yes	5134	5080	54	2.92	0.53	0.39	0.72	< .0001
Postoperative infection								
No	14630	14473	157	3.11	1			
Yes	772	689	83	33.63	10.85	8.21	14.34	< .0001
Gender								
Female	11665	11500	165	4.06	1			
Male	3737	3662	75	6.12	1.30	0.99	1.72	0.064
Age (years)								
50–54	1195	1172	23	6.11	1			
55–64	5970	5855	115	5.90	1.17	0.74	1.85	0.508
65–74	6887	6798	89	3.60	0.69	0.43	1.10	0.118
≥75	1350	1337	13	2.67	0.50	0.25	1.00	0.050
Monthly income (NT$)								
Low-income households	64	62	2	8.72	1			
≤17280	613	608	5	2.38	0.72	0.14	3.78	0.693
17281–22080	11486	11307	179	4.49	1.10	0.27	4.51	0.899
22081–28800	1206	1188	18	4.77	1.28	0.29	5.67	0.743
28801–36300	688	676	12	5.36	1.34	0.29	6.14	0.705
36301–45800	653	643	10	4.40	1.15	0.25	5.39	0.859
≥45801	692	678	14	5.76	1.86	0.41	8.44	0.421
Degree of urbanization in area of residence								
Level 1	3162	3120	42	3.91	1			
Level 2	3974	3912	62	4.65	1.14	0.76	1.69	0.528
Level 3	2265	2233	32	4.21	1.00	0.63	1.6	1.000
Level 4	3099	3040	59	5.43	1.37	0.9	2.08	0.145
Level 5	619	609	10	4.42	1.07	0.53	2.16	0.862
Level 6	1281	1262	19	4.31	0.98	0.56	1.73	0.955
Level 7	1002	986	16	4.39	1.11	0.61	2.01	0.739
CCI score								
0	5293	5222	71	3.97	1			
1	8227	8090	137	4.77	1.07	0.79	1.44	0.669
≥2	1882	1850	32	5.13	1.08	0.70	1.69	0.723
DCSI score								
0	9990	9832	158	4.54	1			
1	2763	2718	45	4.80	1.16	0.82	1.62	0.403
≥2	2649	2612	37	4.27	0.85	0.58	1.23	0.385
Service volume (surgeon)								
Low	919	898	21	6.33	1			
Moderate	4137	4060	77	5.30	1	0.60	1.68	0.998
High	10346	10204	142	4.05	0.88	0.52	1.49	0.628
Service volume (medical institution)								
Low	358	346	12	8.28	1			
Moderate	3612	3542	70	5.25	0.87	0.45	1.70	0.681
High	11432	11274	158	4.15	0.76	0.38	1.52	0.431
Medical institution type								
Medical center	5351	5278	73	3.78	1			
Regional hospital	5798	5699	99	5.17	1.07	0.77	1.48	0.698
District hospital	4253	4185	68	4.72	1.11	0.77	1.60	0.578
Ownership of medical institution								
Public	5245	5162	83	4.63	1			
Private	10157	10000	157	4.49	1.04	0.79	1.37	0.778
Primary diagnosis								
Degenerative arthritis	15040	14810	230	4.46	1			
Rheumatoid arthritis	81	77	4	12.72	2.41	0.86	6.7	0.093
Others	281	275	6	6.04	0.68	0.30	1.54	0.352
Year								
2002 (Ref.)	2138	2085	53	5.19	1			
2003	1987	1952	35	3.96	0.70	0.46	1.08	0.108
2004	1952	1911	41	5.01	0.89	0.59	1.34	0.579
2005	1823	1793	30	4.23	0.74	0.47	1.17	0.198
2006	1584	1555	29	5.35	0.91	0.57	1.44	0.673
2007	1528	1514	14	3.06	0.52	0.29	0.94	0.030
2008	1371	1355	16	4.51	0.77	0.43	1.36	0.360
2009	1117	1104	13	5.39	0.84	0.45	1.57	0.591
2010	914	907	7	4.56	0.76	0.34	1.68	0.492
2011	656	655	1	1.25	0.21	0.03	1.50	0.119
2012	332	331	1	3.80	0.62	0.08	4.50	0.632

† Cox proportional hazard model results

The Cox proportional hazards model was used to examine the related risk factors of revision TKA, and the results are shown in Table 4. After all other variables were controlled, the risk of revision TKA for P4P patients was only 0.53 times that of non-P4P patients (HR = 0.53, 95% CI: 0.39–0.72). Postoperative infection and age were also found to influence revision TKA; patients who had been infected after receiving TKA faced a greater risk of revision (HR = 10.85, 95% CI: 8.21–14.34), whereas patients more advanced in age faced a lower risk than younger patients (HR = 0.50, 95% CI: 0.25–1.00).

## Discussion

This study found that P4P patients faced a lower risk of postoperative infection following TKA. However, after using the Cox proportional hazards model to control related variables, although joining the P4P program was still associated with a lower rate of postoperative infection (HR = 0.91), it was statistically nonsignificant (*p* > 0.05). This was inconsistent with the findings of previous studies which suggest that case management interventions can effectively reduce the risk of postoperative infection [32]. According to the literature, patients with diabetes exhibit relatively more effective blood glucose control after joining P4P programs [21], but the views vary on whether preoperative blood glucose control effectively reduces TKA postoperative infection; some support this notion [10, 33], whereas others do not [34, 35]. Even among the proponents, opinions vary as to what extent blood glucose should be controlled to effectively lower the risk. Nevertheless, a conclusion can be drawn that although participation in a P4P program helps patients with diabetes lower their levels of blood glucose and HbA1c, it is probably not sufficient to facilitate a significant reduction in postoperative infection risk. This accords with this study, in which the P4P program was associated with lowering infection (HR = 0.91), but the result did not reach statistical significance (*p* = 0.277).

In this study, the incidence rate of TKA postoperative infection was highest among patients <55 years old, and the rate dropped as age increased. Presumably, most younger patients were forced to undergo TKA because of secondary arthritis, and as most of them had underwent arthrotomy before, they became a high-risk group for TKA postoperative infection [36–38].

This study also found that male patients faced a 1.51 times higher risk of TKA postoperative infection than female patients, which is corroborated by most related studies. This can probably be attributed to behaviors such as smoking, diet, and personal hygiene [39]. Furthermore, the results of this study also suggest that patients with a lower socioeconomic status face a greater risk of TKA postoperative infection, which is likely the result of lower hygiene habits, less access to medical information [40], and less self-care facility [41, 42]. This echoes the findings of most studies [43].

Regarding the severity of comorbid conditions, this study found that the greater the severity (CCI), the greater the risk of TKA postoperative infection; this was consistent with most other studies [24, 44]. According to the literature, hepatopathy, malignant tumor [25], chronic renal failure, and cardiovascular diseases such as coronary artery disease, valvular heart disease, atrial fibrillation, and heart failure [45] can increase the risk of TKA postoperative infection. All of these items are included in the calculation of the CCI score. Therefore, the association between a higher CCI score and a higher postoperative infection risk is reasonable. Similarly, this study found that the greater the severity of diabetic complications (DCSI), the greater the risk of TKA postoperative infection. Other studies have also found that diabetes complications are a risk factor for TKA postoperative infection [46]; this is likely because diabetic patients with complications have often had diabetes for a longer duration, which weakens their immune systems and makes them more susceptible to infections.

This study found that medical centers, private hospitals, and primary surgeons with high service volume are associated with a lower risk of TKA postoperative infection. The risk was 37% lower with high-service-volume surgeons than their low-service-volume counterparts, perhaps because surgeons with a high service volume are more experienced and well-practiced, allowing them to shorten the duration of operation and thus reduce the risk of infection [47]. The finding that the risk is lower in medical centers than regional hospitals (where the risk is 1.9 times higher) contradicts some studies that claim because complicated cases are mostly sent to medical centers, they have a higher incidence rate of infection [47]. This may be attributed to the growing popularity of and proficiency in TKA in Taiwan, which contributes to the reduction of selective referrals; moreover, medical centers tend to be better equipped. These reasons may explain why medical centers may have lower postoperative infection risks [48]. Compared with public hospitals, the risk of postoperative infection was found to be 33% lower in private hospitals. However, this could be the result of sending patients with a higher severity or poorer personal hygiene to public hospitals. Other studies have found that patients receiving TKA for traumatic arthritis face a higher risk of postoperative infection, which corroborates this study’s findings that diabetic patients receiving TKA for reasons other than degenerative and rheumatoid arthritis faced a higher risk of postoperative infection [37].

The Cox proportional hazards model results indicated that P4P patients faced a lower risk of revision TKA (HR = 0.53). Three possible reasons are: (1) The P4P program improves physicians’ and patients’ adherence to instructions, which contributes to more effective blood glucose control [49]. Although joining the P4P program did not significantly reduce TKA postoperative infection, it did result in a tendency toward that goal. (2) Some studies have suggested that joining a P4P program can improve patients’ rate of regular return visits, improving patients’ understanding of their conditions. Moreover, joining the program allows patients to receive significantly improved medical attention (4.27 times better than otherwise) [50], enabling early detection of worn linear and loosened prosthetic devices to prevent revision TKA. (3) Because body weight has been identified as an independent factor causing revision TKA [51], multifunctional healthcare teams that include physicians, nutritionists, and health education specialists are able to provide more comprehensive care (including weight and blood glucose control) than others.

The 55–64 age group had the highest risk of revision TKA, after which the risk decreased as age increased. The literature corroborates this; for every 10 years of increased age, the risk decreases by 40% [52]. This is assumed to result from the greater activity of younger people, which can easily result in worn pads and loosened prosthetic devices; moreover, older people are also less suitable surgical candidates for health reasons [52, 53]. Regarding the role of gender, some researchers believe that because men have higher body weight and more active, they are more susceptible to infections, leading to a greater risk of revision TKA [54]however, some researchers disagree and assert that women are at greater risk [55]. The results of this study found that although male patients exhibited a higher tendency of revision TKA, it was statistically nonsignificant (*p* > 0.05).

This study also found that the risk of revision TKA was 10.85 times higher for patients with postoperative infection than those without. If an infection is acute, even if the site can be cleared up quickly, the chance of retaining the prosthetic device is between 31% and 100%; if the infection is chronic, the chance is reduced to 28%–62% [56]. Therefore, infection often results in the removal of the artificial knee for revision TKA, which is why postoperative infection can increase the risk of revision TKA. Additionally, the bivariate analysis results indicated that a primary surgeon and medical institution with a high service volume significantly lower the risk of revision TKA. This is probably explained by the greater experience and proficiency of such surgeons and institutions, which helps reduce postoperative infection [57] and thus the risk of revision TKA. However, in the multivariate analysis, the association between service volume and reduced risk was nonsignificant. This is probably because the explanatory power of high service volume (of both the surgeons and the medical institutions) has been diluted by postoperative infection.

There were some limitations in our study. The NHI database provided secondary data concerning the medical expense declarations of the institutions involved. In view of the restrictions posed by the default variables in the data format, not all of the patients’ related characteristics (e.g. lifestyle, BMI, and health behaviors (smoking status) and lab data (HgbA1C levels)) could be included for analysis, nor could the types of prosthetic device or the surgical methods be discerned in the data. We utilized administrative data and ICD-9 codes to account for post-operative infection rates. Administrative data may not be as accurate and clinical data in capturing post-operative complications. Furthermore, the data were only tracked for 11 years, which is too short because this is within the service life of most of the artificial knees; the results would have been more meaningful if a longer tracking duration had been implemented.

The Taiwanese health care system is different from other health care systems in the world. Taiwan P4P program has different designs from other countries, so our research results may be limited.

## Conclusions

Joining the P4P program is associated with a tendency of lower TKA postoperative infection rate, as well as a lowered risk of revision TKA. Being male, younger, or with low-income; having multiple comorbid conditions or greater diabetic severity (DCSI); receiving care at regional or public hospitals; or not being diagnosed with degenerative or rheumatoid arthritis were identified as factors for a higher risk of TKA postoperative infection. By contrast, a high service volume of the primary surgeon was associated with a lower risk of TKA postoperative infection. Furthermore, patients with postoperative infection had a greater risk of revision TKA, unlike older patients, whose risk of revision TKA was lowered with the advance of age. For patients with diabetes who are about to undergo TKA, participation in coordinated medical care services (such as the P4P program) is highly recommended, because the comprehensive care it provides can help reduce the risk of TKA postoperative infection and revision.

## Abbreviations

NHI: National health Insurance
NHIRD: National Health Insurance Research Database
CI: confidence interval
CCI: Charlson Comorbidity Index
WHO: World Health Organization
ICD-9-CM: International Classification of Diseases, Ninth Revision, Clinical Modification
DCSI: diabetes complication severity index
DM: Diabetes Mellitus
Adj. HR: adjusted hazards ratio
NTD: New Taiwan Dollar
P4P: pay for performance
TKA: total knee arthroplasty
